# Sub-chronic toxicity of the aqueous leaf extract of *Ocimum lamiifolium* Hochst. ex Benth on biochemical parameters and histopathology of liver and kidney in rats: in vivo and *in- silico* toxicity studies

**DOI:** 10.1186/s12906-023-03863-7

**Published:** 2023-02-02

**Authors:** Fentahun Adane, Wubshet Assefa, Mamaru Bitew Alem, Megbar Dessalegn

**Affiliations:** 1 Department of Biomedical Sciences, School of Medicine, Debre Markos University, Debre Markos, Ethiopia; 2 Department of Pathology, School of Medicine, Debre Markos University, Debre Markos, Ethiopia; 3 Department of Chemistry, College of Natural and Computational Sciences, Debre Markos University, Debre Markos, Ethiopia; 4 Department of Surgery, School of Medicine, Debre Markos University, Debre Markos, Ethiopia

**Keywords:** *Ocimum lamiifolium*, Liver; Kidney, Sub-chronic toxicity, Biochemical profile, In-silico toxicity study, And Wistar Rats

## Abstract

**Background:**

The aerial part of *Ocimum lamiifolium* is commonly used in Ethiopian traditional medicine. Although this plant is mostly used in traditional medicine, its safety profile has not been documented yet. The aim of this study was to assess the sub-chronic toxicity of *O. lamiifolium* aqueous extract in rats and to determine the toxicity profile of GC–MS identified bioactive compounds obtained from essential oil of *O. lamiifolium* using in silico toxicity methods.

**Methods:**

Eighty rats (40 male and 40 female) were randomly assigned to four groups of ten rats per sex/group. For 90 days, Groups I-III received 200, 400, and 600 mg/kg bw of aqueous extract of *O. lamiifolium*, respectively. Distilled water was given to Group IV (control). Clinical observations, food intake, and rat weight were all recorded during the experiment. In addition, several biochemical parameters, organ weight, and histology of the liver and kidney were all evaluated. For the *in-silico* toxicity study, GC–MS identified bioactive compounds in *O. lamiifolium* essential oil were obtained from published articles. The compounds two-dimensional structures were constructed using Chemdraw. The two-dimensional structures were converted into a simplified molecular input line entry system (SMILES) using the Swiss ADMET web tool. Furthermore, the toxicity parameters were predicted using the *ProTox II* server.

**Results:**

The administration of an aqueous extract of *O. lamiifolium* leaves significantly (*p* < 0.05) reduced the test animals' food intake and body weight gain. In the high dose (600 mg/kg bw) treated group, the serum alanine aminotransferase, aspartate aminotransferase, and alkaline phosphatase levels were significantly increased (*p* < 0.05). In female rats given 600 mg/kg bw of *O. lamiifolium*, the levels of serum urea were also increased. In addition, rats given 600 mg/kg bw had significantly lower blood glucose levels than the control group (*p* < 0.05). Doses up to 400 mg/kg bw didn’t bring a significant change to the histology of the liver. However, in the high dose (600 mg/kg bw) treated group, some female rats' livers showed mild sinusoidal and central vein dilatation, as well as parenchymal necrosis. our findings showed that all compounds derived from the essential oil of *O. lamiifolium* showed no mutagenicity or cytotoxicity. However, 30% of the compounds tested were hepatotoxic, 20% carcinogenic, and 20% immunotoxin.

**Conclusion:**

Our findings showed that oral administration of *O. lamiifoliums* aqueous extract up to a dose of 400 mg/kg bw is not toxic. However, high-dose (600 mg/kg bw) significantly affected the food consumption and weight gain of the experimental rats and the serum concentration of some liver and kidney enzymes were also significantly increased. Additionally, a considerable proportion of the tested compounds were predicted to be hepatotoxic, carcinogenic and immunotoxin. Furthermore, before employing *O. lamiifolium* preparations as drugs, a chronic toxicity research on the essential oil as well as its components that exhibited toxicity in the in-silico toxicity study is needed. Finally, use high doses of *O. lamiifolium* leaves with caution.

**Supplementary Information:**

The online version contains supplementary material available at 10.1186/s12906-023-03863-7.

## Introduction

The genus *Ocimum* (Lamiaceae) is widespread and can be found in a variety of habitats, with some species being grown in temperate climates [1]. Among the 12 *Ocimum* species found in Ethiopia [2], *Ocimum Basilicum* and *Ocimum lamiifolium* are most commonly used in herbal medicine and as culinary herbs. The plant's scientific name is *Ocimum lamiifolium*, and it is also known as 'Damakessie' in Amharic [2].

Traditional medicine is common in Africa, Asia, and other developing nations, especially in rural regions, to maintain their health and treat a variety of ailments. According to the World Health Organization (WHO), approximately 80% of people in developing countries rely on traditional medicine for primary health care, with plant extracts accounting for a significant proportion of this magnitude [3].

*O. lamiifolium* has been used in Ethiopian traditional medicine to treat a variety of ailments. The leaf of *O. lamiifolium* is traditionally used for the treatment of diarrhoea, stomach disorders, abdominal pains [4–7], headache and fever [8], cough [9], malaise, and pain [10]. It is also used to treat colds, measles, and eye infections [11], as well as a mosquito repellent [7, 12]. In vivo and in vitro studies on the antimalarial, insecticidal, analgesic, anti-inflammatory, hepatoprotective, antipyretic, antidiarrheal, and antioxidant activities of *O. lamiifoliums* yielded positive results so far [13–20].

*O. lamiifolium* contains approximately 0.22% (v/w) volatile oil with a colourless, pungent odour. The essential oil of *O. lamiifolium* contains chemical compounds such as alkaloids, sterols, carbohydrates, glycosides, tannins, flavonoids, bornyl acetate, p-cymene, camphene, a-pinene, and sabinene [19, 21–24]. These chemical components have antimicrobial, antibacterial, and antifungal properties [7, 10, 14].

Although the leaves of *O. lamiifolium* have many pharmacological properties, they are also well-known, praised, and widely used home remedies. However, the toxicity of *O. lamiifolium* has not been studied to determine its potential clinical application. This study, therefore, aimed to determine the in vivo sub-chronic toxicity effects of the plant extracts in rats and the *in-silico* toxicity profiles of compounds extracted from *O. lamiifolium*.

## Materials and methods

### Plant material

Fresh *O. lamiifolium* leaves were collected from their common natural habitats in Enarj Enawga District, East Gojjam Zone, Amhara Region, Ethiopia in October 2021. Enarj Enawga is approximately 195 kms southeast of Bahir Dar, the capital city of the Amhara National Regional State, and 291 kms north of Addis Ababa, Ethiopia's capital city [25]. The identification and authentication of the leaf of the plant was done by a botanist from the Department of Biology Debre Markos university, and registered with the voucher (No. HH-001) for future reference. The fresh leaves of *O.* were shade dried at room temperature (28 ± 2 °C) for 30 days.

#### Extraction of plant material

In an orbital shaker, 700 g of powdered *O. lamiifolium* leaves were macerated in distilled water for 72 h [26]. After that, the supernatant was decanted and filtered through 0.1 mm^2^ mesh gauze. Finally, the filtrate was lyophilized to produce 60.5 g (10.08 percent w/w) of brownish extract, which was kept at 4 °C in a desiccator until use.

#### Experimental animals

The experimental animals were young male and female (nulliparous) Wistar albino rats weighing 220 to 260 g and aged 10 to 12 weeks. The experimental rats were obtained from the Ethiopian Public Health Institute's (EPHI) animal breeding unit. Rats were acclimatized for a week before being housed in the laboratory of EPHI's Traditional and Modern Medicine Research Directorate (TMMRD). The rats were kept in a stainless-steel cage at room temperature (23 ± 3 °C) with a relative humidity of 50% under a controlled 12-h light–dark cycle. A standard laboratory diet and an unlimited supply of drinking water were provided throughout the experiment. Eighty rats (40 male and 40 female) were randomly assigned to four groups of ten rats per sex/group. A computer-based random order generator was used to randomize the rats. Based on a previous study, rats in groups I to III received 200, 400, and 600 mg/kg bw of body weight of the aqueous *O. lamiifolium* leave extract, respectively [14]. Rats in the fourth (negative control) group were given distilled water (1 mL/100 g body weight). A gavage was used to administer the extracts to the rats in the treatment group and distilled water to control groups for 90 days. The overall experimental procedures followed the Organization for Economic Cooperation and Development (OECD) guidelines [27]. The data collector, who was unaware of the treated and control rats, measured the outcomes blindly. All procedures used in this study were approved by Debre Markos University, School of Medicine ethical review committee on September 18, 2021, with a letter number M/R/CS/61/03/21.

### Clinical examination, measurement of body weight and food intake

A general clinical observation was performed during the treatment period every morning at 8:00 AM. Before and after dosing, any signs of toxicity were recorded, including changes in the skin, motor and sensory function, unusual respiratory patterns, and self-mutilation. Every day, all rats were monitored for severe toxicity, morbidity, and mortality. The weight of rats was measured on the first day of administration, weekly thereafter, and at necropsy, and the weight gain was calculated. In addition, food intake was monitored daily throughout the experiment.

### Liver and kidneys weight

Rats were fasted overnight at the end of the treatment period (90 days), before being anesthetized with an intraperitoneal injection of pentobarbital (150 mg/kg bw) [28]. The liver and the kidneys were harvested from the experimental animals and carefully dissected free of fat and examined for any gross pathological alterations after a longitudinal incision was made through the anterior abdominal wall. An electric balance (model of Mettler Toledo ME204E ME Series) sensitive to 0.0001 g was used to weigh the liver and the kidneys. The relative organ weight was calculated by dividing the absolute organ weight by the final rat weight and multiplying it by 100.

### Clinical chemistry analysis

A blood sample was taken for clinical chemistry analysis before dissecting fasted rats. A total of 60 mL of blood was collected via cardiac puncture at the end of the treatment period. For an hour, the blood was placed in a plane test tube. To obtain serum, the blood was centrifuged for 10 min at 3500 rpm in an electrical centrifuge. The serum was withdrawn with a micropipette and stored in a vial. Finally, the serum was immediately analysed by an automated clinical chemistry analyser (AUTO LAB 18, clinical chemistry analyser Italy), and the values of the following enzymes were determined to test renal and hepatic functions: alanine aminotransferase (ALT), aspartate aminotransferase (AST), alkaline phosphatase (ALP), urea, creatinine, total protein, albumin, glucose, and total cholesterol.

### Histopathology of the liver and the kidneys

To assess treatment-related histopathologic changes, liver and kidney samples were obtained. The tissue samples were fixed in 10% formalin overnight. Tissue processing and staining protocols were based on Bancroft's theory and the practice of histological techniques [29]. In a nutshell, the tissues were dehydrated using an ascending series of alcohol (40%, 50%, 70%, 80%, 90%, and 100%). To clean the tissues, xylene was used. The tissues were then embedded after being impregnated with melted paraffin wax. To stain the tissues, a five-millimetre section was cut. The slides with the ribbons were placed in a hot oven (40- 45 ^0^C) for 20 -30 min to remove excess wax and facilitate tissue adherence on the slides. The slides were dewaxed with xylene (I, II, and III) for five minutes each, dehydrated with descending series of alcohol (absolute alcohol I, absolute alcohol II, 90% alcohol, 80% alcohol, and 70% alcohol) for two minutes each, and washed with running tap water for two minutes. The slides were then stained for 6- 10 min with Harris hematoxylin, cleaned with running tap water for 10 min, immersed in acid alcohol for 2–3 s, and counterstained with eosin Y for 1- 2 min. The stained slides were dehydrated in an ascending series of alcohol (80%, 95%, absolute alcohol I, and II) for two minutes each before being cleared with xylene I, II, and III for two minutes each. Finally, the cleared slides were mounted with Dibutylphthalate Polystyrene Xylene (DPX) and a cover slip was applied [29]. A pathologist used a binocular light microscope to perform the detailed microscopic examination for any treatment-related changes after the sides were dried. The histology of the liver and the kidney in the treatment and control groups was compared. Following microscopic examination, representative photomicrographs were captured using an automated built-in digital microscope camera (Leica EC4, Germany) with objective lens magnifications of 10 × and 40x, and total magnifications of 100 × and 400x, respectively.

### In silico toxicity prediction

In the previously published article, compounds found in the essential oil of *O. lamiifolium* were identified using GC–MS [21]. Chemdraw (8.0) [30] was used to create two-dimensional structures (Table 1). The Swiss ADME web tool was used to convert the two-dimensional structures into a simplified molecular-input line input system (SMILES) that can be analyzed by servers for toxicity prediction [31].

**Table 1:** Structure and SMILES of major compounds of O. *lamiifolium*

Compounds	Chemical structure	SMILES
Linalol		CC(C) = CCCC(C)(O)C = C
1-Octen-3-ylpropionate		CCCCCC(C = C)C(C)C(O) = O
3,7,11-Trimethyl-(E, E)-2,6,10-dodecatrienal		CC(C) = CCC\C(C) = C\CC\C(C) = C\C = O
7-Methylene bicyclo[3,3,1]nonan-3-ol		OC1CC2CC(C1)CC(= C)C2
2,4,5,6,7,7-Hexahydro-4,7-methano-1H-indene		C1CC2C3CCC(C3)C2 = C1
δ-Cadinene		[H][C@@]12C = C(C)CCC1 = C(C)C = C[C@H]2C(C)C
2-Methoxy-4-(1-propenyl)-phenol		COC1 = CC(\C = C\C) = CC = C1O
α-Terpineol		CC1 = CCC(CC1)C(C)(C)O
3-Octanol		CCCCCC(O)CC
2-Methylphenyl p-methoxybenzoate		COC1 = CC = C(C = C1)C(= O)OC1 = CC = CC = C1C

The toxicological endpoints (hepatotoxicity, carcinogenicity, immunotoxicity, cytotoxicity, and mutagenicity) and level of toxicity (LD_50_, mg/kg) of the investigated compounds were determined using the *ProTox-II* server [32, 33].

### Data processing and analysis

A statistical package for social science (SPSS) version 24 was used to analyse the data, and the results were expressed as the mean of the values ± Standard deviation (SD). A one-way analysis of variance (ANOVA) was performed, followed by Turkey (to test any difference among the four groups) and Dunnett (to test the difference between control and treated groups) post hoc tests. P values ≤ 0.05 were deemed statistically significant.

## Results

### Clinical examination, measurement of body weight and food intake

Daily cage-side clinical observations were performed before and after dosing periods. The results of these records revealed no changes in skin, hair, or mucus membranes. Furthermore, no changes in respiratory pattern, motor activity, self-mutilation, or other toxicity signs were observed. There were no deaths recorded during the experiment, indicating that the rats tolerated the 90-day oral administration of the test *O. lamifolium* extracts well. Table 2 shows the levels of food intake and weight gain of the test animals. Male rats challenged with a high dose of the O. lamifolium extract had significantly lower food intake (195.2 ± 12.1 g) than those in the control group (220.1 ± 13.3 g). In addition, the rats' weight gain was significantly lower in a high-dose treatment group (60.0 ± 11.1 g) as compared to the control (83.7 ± 5.5 g) low-dose treated groups.

**Table 2:** Weight gain and food intake of rats treated with the aqueous extract of *O.lamiifolium*

Sex	Food intake and weight gain	Groups with respective dose of compounds			
		**Group I (200 mg/ Kg)**	**Group II (400 mg/ Kg)**	**Group III (600 mg/ Kg)**	**Group IV Control**
Male	Food intake (g) *n* = 10	213.6 ± 15.3	208.2 ± 10.1	195.2 ± 12.1^a^	220.1 ± 13.3
	Weight gain/ rat (g)	81.1 ± 5.0	75.0 ± 11.7	60.0 ± 11.1^b^	83.7 ± 5.5
Female	Food intake (g) *n* = 10	152.4 ± 7.1	150.4 ± 7.2	137.3 ± 4.2^c^	151.5 ± 11.1
	Weight gain/ rat (g)	44.9 ± 6.1	39.5 ± 5.7	30.9 ± 6.7 ^b^	± 5.3

Results are expressed as mean ± SMD

^a^ Significantly different from the control group

^b^ Significant difference with groups I and IV

^c^ Significant difference with all the other groups

(*p* < 0.05). One-way ANOVA

Female rats given 600 mg/kg bw of the *O. lamifolium* aqueous extract had lower food intake (137.3 ± 4.2 g) compared to the control (151.5 ± 11.1 g) and the other treatment groups. Similarly, female rats given 600 mg/kg bw of *O. lamifolium* aqueous extract gained significantly less weight (30.9 ± 6.7 g) than the control (44.1 ± 5.3 g) and low dose treated groups.

### Weight and gross examination of the liver and kidneys

**Fig. 1:**
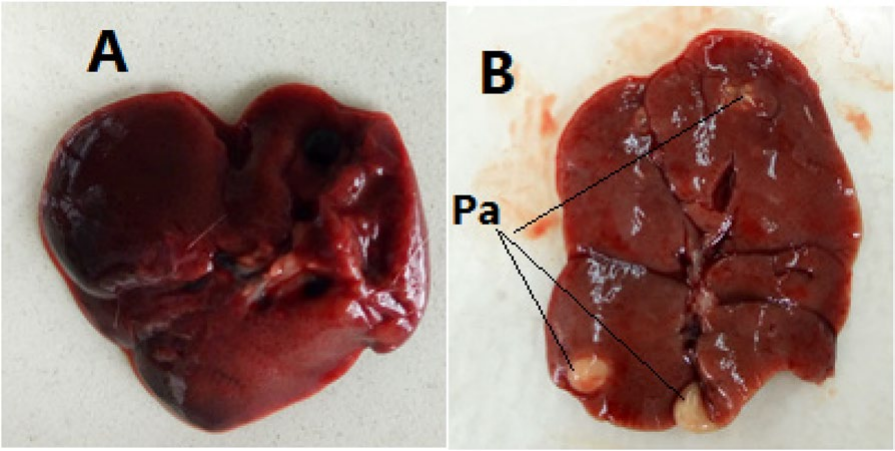
Gross structure of the rat liver. **A** Normal gross structure of the liver in the control group and (**B**) gross pyogenic abscess (Pa) found in the liver of rats in the high dose (600 mg/kg bw) treated group

**Fig. 2:**
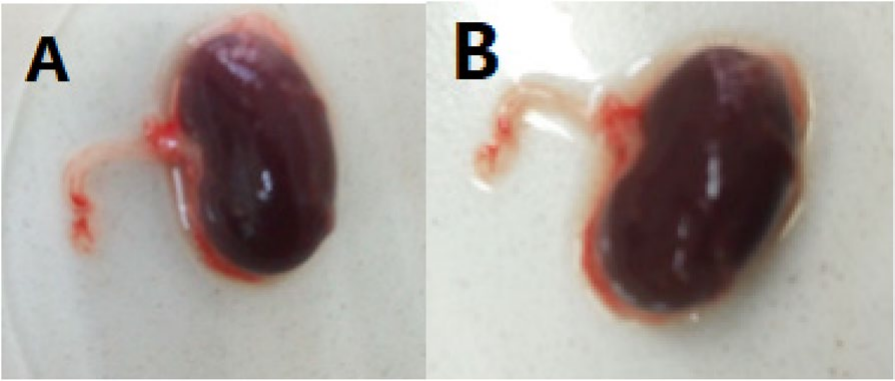
Gross structure of the rat kidneys. **A** Photographs of rat kidneys from the control group and (B) the high dose (600 mg/kg bw) group with normal gross structures after administration of *O.lamiifolium* aqueous extract (600 mg/kg bw)

A macroscopic examination of the liver and the kidneys was performed during necropsy. Pyogenic abscess gross abnormalities of the liver were observed in some female rats in the high doses (600 mg/kg bw) treatment group (Fig. 1B) compared to the control group (Fig. 1A). However, no gross abnormalities in the colour, texture, size, or shape of the kidneys were observed across the experimental groups (Fig. 2 A & B).

In male rats of the high dose group (600 mg/kg bw), the weight of the liver was significantly increased (5.2 ± 0.3) compared to the control group (3.5 ± 0.7). Similarly, the relative liver weight in the high dose (600 mg/kg bw) treatment group was significantly increased (5.0 ± 0.6) than the control group (3.3 ± 0.5). However, in both male and female rats, there was no significant difference in the relative weight of the kidneys between the control and treatment groups (Table 3).

**Table 3:** Relative organ weight of rats treated with the aqueous extract of *O.lamiifolium*

Gender of the rats	Organ weight (g)	Groups			
		Group I 200 mg/ kg	Group II 400 mg/ kg	Group III 600 mg/ kg	Group IV Control
Male	Liver	3.3 ± 1.2	2.4 ± 0.2	5.2 ± 0.3 ^a^	3.5 ± 0.7
	Kidney	0.4 ± 0.2	0.4 ± 0.1	0.4 ± 0.2	0.4 ± 0.2
Female	Liver	3.6 ± 0.6	3.7 ± 0.5	5.0 ± 0.6 ^a^	3.3 ± 0.5
	Kidney	0.5 ± 0.1	0.4 ± 0.1	0.4 ± 0.1	0.4 ± 0.1

Results are expressed as mean ± standard mean deviation (SMD)

^a^ significantly different from group IV

### Biochemical profiles of the rats

Male rats which were given a high dose of *O. lamiifolium* leave extract (600 mg/kg bw) had significantly higher levels of serum ALT (69.0 ± 8.6), AST (184.4 ± 18.1), and ALP (106.2 ± 5.4) than those in the control group ALT (47.4 ± 6.4), AST (137.7 ± 20.5) and ALP (78.2 ± 2.0). Serum ALP levels were significantly higher in rats treated with 600 mg/kg bw of the extract compared to those in low dose treated and control groups. Furthermore, rats given 600 mg/kg bw of *O. lamiifolium* extract had lower serum glucose levels than the control group. However, there was no significant difference between the treatment and control groups in terms of the other liver and kidney function tests (*P* > 0.05) (Table 4).

**Table 4:** Biochemical profile of male rats treated with the aqueous extract of *O. lamiifolium*

Tests	Group			
	Group I 200 mg/ kg	Group II 400 mg/ kg	Group III 600 mg/ kg	Group IV Control
ALT (U/L)	47.8 ± 12.3	47.7 ± 4.0	69.0 ± 8.6^a^	47.4 ± 6.4
AST (U/L)	129.2 ± 10.8	118.4 ± 9.4	184.4 ± 18.1^b^	137.7 ± 20.5
ALP (U/L)	76.2 ± 1.4	80.0 ± 7.0	106.2 ± 5.4^c^	78.2 ± 2.0
Urea (mg/dL)	48.6 ± 5.1	44.1 ± 2.5	42.5 ± 7.4	49.7 ± 3.0
Creatinine(mg/dL)	0.30 ± 0.0	0.32 ± 0.0	0.32 ± 0.0	0.35 ± 0.0
Albumin (g/dL)	4.3 ± 0.1	4.4 ± 0.1	4.4 ± 0.1	4.1 ± 0.2
Total protein (g/dL)	5.9 ± 0.1	5.9 ± 0.1	6.1 ± 0.2	6.0 ± 0.1
Total cholesterol (mg/dL)	40.7 ± 2.3	41.5 ± 2.2	42.5 ± 2.5	38.7 ± 1.5
Glucose (mg/dL)	106.8 ± 9.2	96.4 ± 14.8	83.4 ± 10.5^a^	123.5 ± 11.7

Results are expressed as mean ± SMD. For all p-value was < 0.05, One-Way ANOVA. *ALT* alanine aminotransferase, *AST* aspartate aminotransferase, *ALP* alkaline phosphatase

^a^ significantly different from the control group (Dunnett test)

^b^ significant difference with all the other groups

^c^ significant difference with groups I and IV

In female rats, rats treated with 600 mg/kg bw of the *O. lamiifolium* extract had a significantly higher level of ALT (78.7 ± 4.7) compared to rats in the control group (54.4 ± 13.7) and the low dose treated groups. Furthermore, serum AST, ALP, and urea levels were significantly higher in the high-dose treated groups compared to the control and low-dose treated groups. Blood glucose level in rats given 600 mg/kg bw of the extract significantly lower (81.0 ± 12.0) than the rats in the control group (120.0 ± 15.0) (Table 5).

**Table 5:** Biochemical profile of Female rats treated with the aqueous extract of *O. lamiifolium*

Tests	Group			
	Group I 200 mg/ kg	Group II 400 mg/ kg	Group III 600 mg/ kg	Group IV Control
ALT (U/L)	54.2 ± 7.3	63.0 ± 4.0	78.7 ± 4.7^a^	54.4 ± 13.7
AST (U/L)	160.1 ± 8.7	164.2 ± 1.2	201.0 ± 4.1^a^	160.9 ± 8.4
ALP (U/L)	105.0 ± 4.6	107.2 ± 6.5	161.3 ± 8.2^a^	101.6 ± 4.1
Urea (mg/dL)	50.1 ± 2.3	54.3 ± 6.8	71.2 ± 10.0^a^	49.9 ± 4.6
Creatinine (mg/dL)	0.3 ± 0.0	0.4 ± 0.0	0.4 ± 0.0	0.3 ± 0.1
Albumin (g/dL)	3.7 ± 0.1	3.3 ± 0.2	3.4 ± 0.2	3.8.0 ± 0.3
Total protein (g/dL)	4.6 ± 0.5	4.4 ± 0.1	4.4 ± 0.1	4.9 ± 0.5
Total cholesterol (mg/dL)	68.6 ± 3.5	69.5 ± 5.8	69.7 ± 5.7	72.8 ± 8.4
Glucose (mg/dL)	101.5 ± 7.8	102.2 ± 8.2	81.0 ± 12.0^b^	120.0 ± 15.0

Results are expressed as mean ± SMD. For all *p* < 0.05, One-Way ANOVA. ALT: alanine aminotransferase, AST: aspartate aminotransferase, ALP: alkaline phosphatase

^a^ significant difference with groups I and  IV

^b^ significantly different from the control group (Dunnett test)

### Histology of the liver and the kidneys

**Fig. 3:**
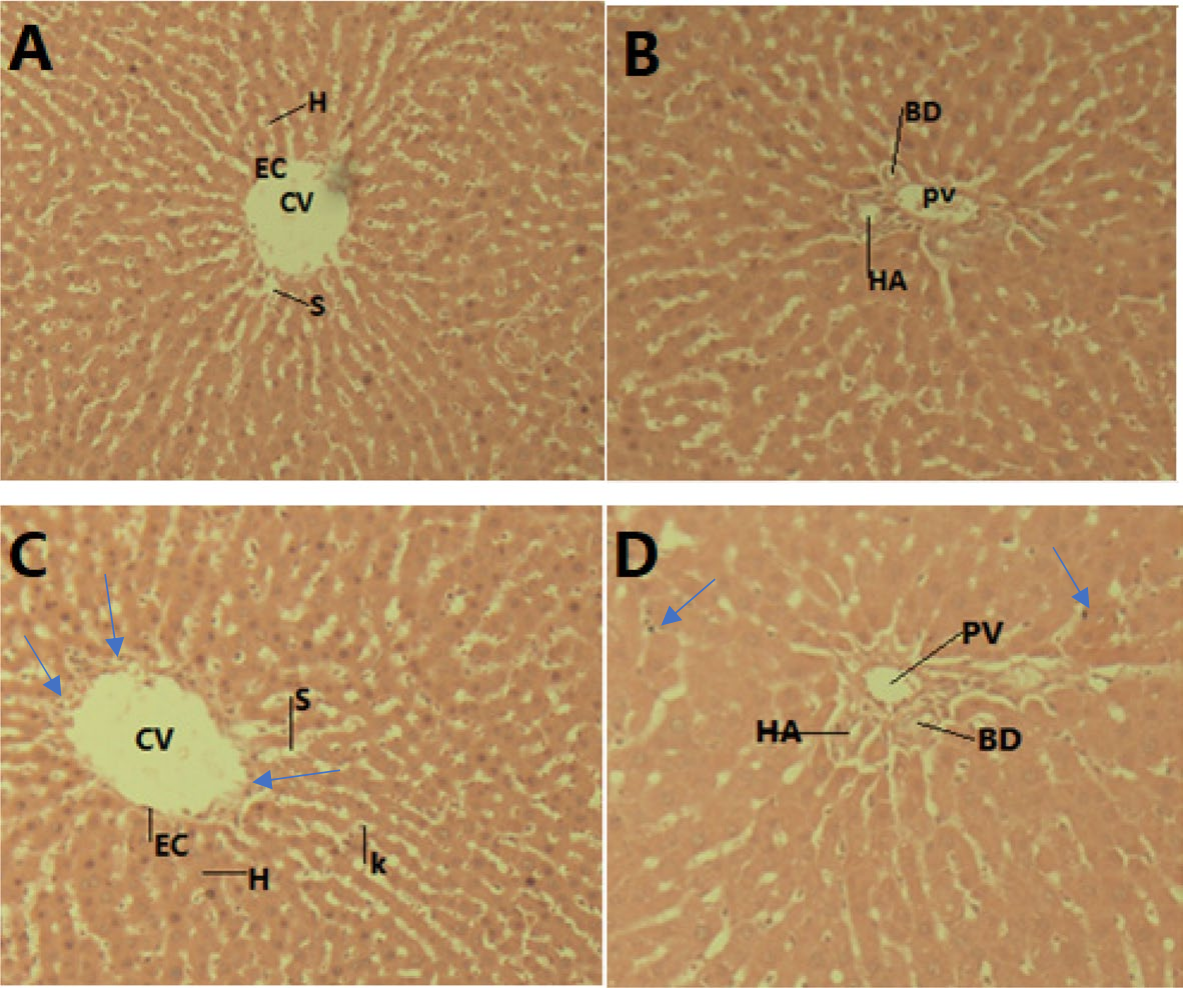
Microscopic structure of the rat liver. **A** and (**B**): Photomicrographs of liver sections of control rats; (**C**) And (**D**): liver sections of rats treated with 600 mg/kg bw of aqueous extract of *O. lamiifolium*; CV = central vein, EC = endothelial cells, H = hepatocytes, K = Kupfer cells, S = sinusoids, BD = bile duct, HA = hepatic artery, and PV = portal vein

The administration of *O. lamiifolium* aqueous extract for 90 days did not result in a significant change in hematoxylin and eosin (H & E) stained histology of the liver. Its microscopic structures includingthe portal triad, bile duct system, hepatocytes, and sinusoids, appeared normal (Fig. 3A & B). A few female rats treated with 600 mg/kg bw of the *O. lamiifolium* extract had only mild sinusoidal dilatation, central vein dilatation, and parenchymal necrosis changes in their livers (Fig. 3C & D). Interestingly, any dose of the plant extract didn’t bring any effect on the histology of the kidneys.

**Fig. 4:**
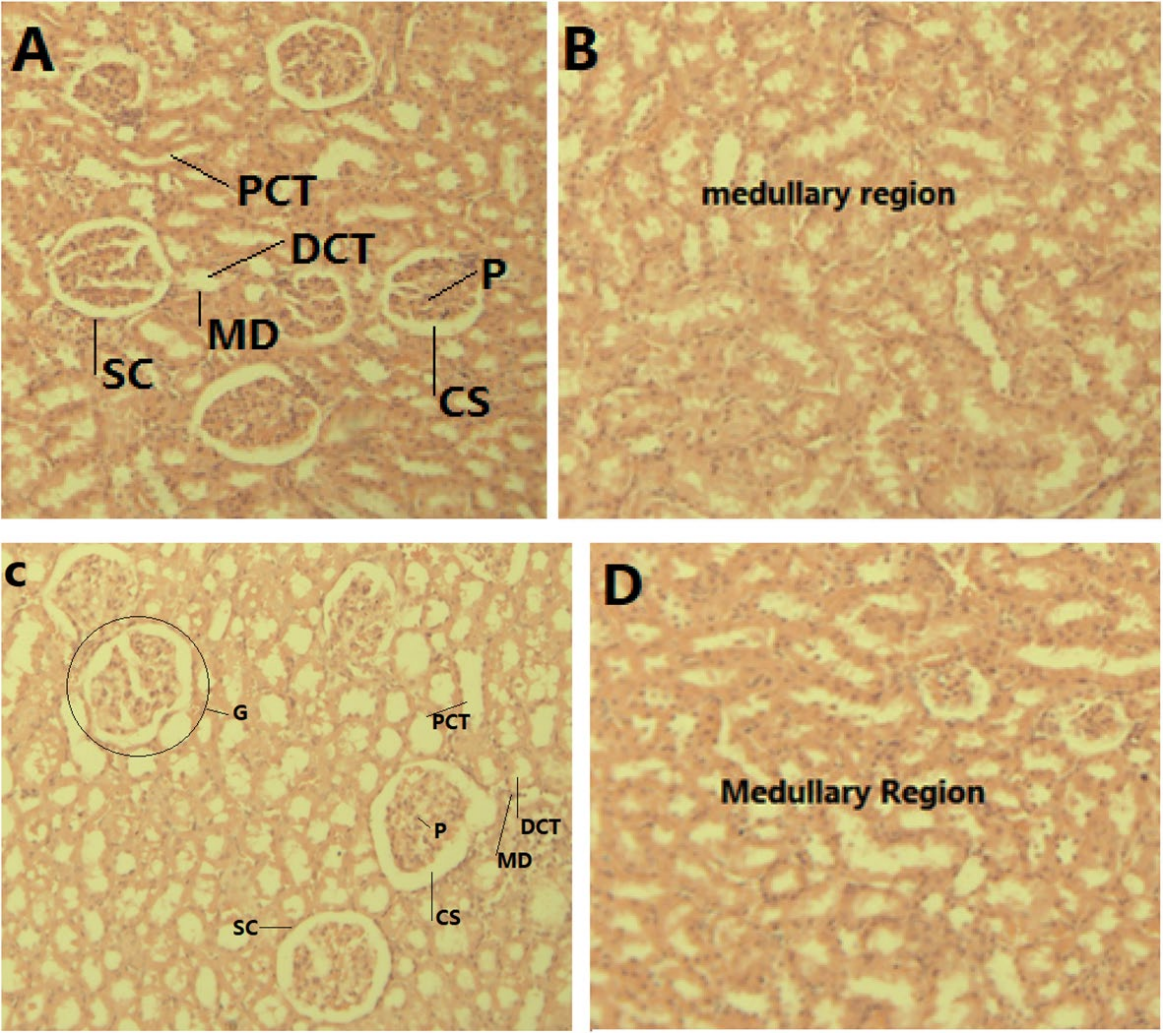
Microscopic structure of the rat kidneys. **A** and (**B**): Photomicrographs of the kidney sections of control rats, (**C**) and (**D**): kidney sections of rats treated with 600 mg/kg bw of aqueous extract of *O.lamiifolium*; PCT = proximal convoluted tubule, DCT = distal convoluted tubule, MD = macula densa, G = glomerulus, CS = capsular space, SC = squamous cell, and P = podocyte

Additionally, the glomerular capillaries, bowman's capsule, afferent and efferent arterioles, and renal tubes showed no structural changes across all groups (Fig. 4).

### In-silico toxicity prediction of major compounds of O.lamiifolium

The toxicity profile of essential oil compounds was also evaluated using the *ProTox-II* server. Toxicity and toxicological endpoint findings revealed that all compounds derived from *O. lamiifolium* essential oil were free of cytotoxicity and mutagenicity. In terms of immunotoxicity parameters, most of the compounds (80%) had no toxicity. Immunotoxin compounds include *δ* -cadinene and *trans*-2-methoxy-4-(1-propenyl)-phenol, which have prediction probabilities of 0.85 and 0.67, respectively. Similarly, the majority of the compounds (70%) tested were inactive for carcinogenicity. However, 30% of the compounds tested were active for carcinogenicity (linalol, 2,4,5,6,7,7-hexahydro-4,7-methano-1 h-indene, and 2-Methoxy-4-(1-propenyl)-phenol), with prediction probabilities of 0.71, 0.68, and 0.65, respectively). Furthermore, the majority of the compounds (70%) did not exhibit hepatotoxicity. However, 30% of the compounds tested were toxic to the liver. The hepatotoxic compounds were 1-octen-3-ylpropionate (0.73), 2-methoxy-4-(1-propenyl)-phenol (0.67), and 2-methylphenyl p-methoxybenzoate (0.62). Finally, from the total 10 compounds, predicted LD_50_ showed that 5 compounds (50%) have toxicity class four (300 < LD50 ≤ 2000) and 5 compounds (50%) have toxicity class five (2000 < LD50 ≤ 5000) (Table 6).

**Table 6:** *In-silico* toxicity prediction of compounds from the essential oil of *O. lamiifolium*

No	Compounds	*Insilco* Toxicity											
		**Hepatotoxicity**		**Carcinogenicity**		**Immunotoxicity**		**Mutagenicity**		**Cytotoxicity**		**LD**_**50**_** values**	**T. class**
		**Toxicity**	**Prob**	**Toxicity**	**Prob**	**Toxicity**	**Prob**	**Toxicity**	**Prob**	**Toxicity**	**Prob**		
1	Linalol	Inactive	0.76	Active	0.71	Inactive	0.99	Inactive	0.95	Inactive	0.82	2200 mg/kg	5
2	1-Octen-3-ylpropionate	Active	0.73	Inactive	0.67	Inactive	0.86	Inactive	0.96	Inactive	0.78	2610 mg/kg	5
3	3,7,11-Trimethyl-(E, E)-2,6,10-dodecatrienal	Inactive	0.69	Inactive	0.87	Inactive	0.99	Inactive	0.98	Inactive	0.82	1300 mg/kg	4
4	7-Methylene bicyclo[3,3,1]nonan-3-ol	Inactive	0.73	Inactive	0.70	Inactive	0.96	Inactive	0.54	Inactive	0.82	2000 mg/kg	4
5	2,4,5,6,7,7-Hexahydro-4,7-methano-1H-indene	Inactive	0.73	Active	0.68	Inactive	0.97	Inactive	0.64	Inactive	0.77	5000 mg/kg	5
6	δ-Cadinene	Inactive	0.76	Inactive	0.73	Active	0.67	Inactive	0.64	Inactive	0.73	4390 mg/kg	5
7	2-Methoxy-4-(1-propenyl)-phenol	Active	0.67	Active	0.65	Active	0.85	Inactive	0.97	Inactive	0.85	1560 mg/kg	4
8	α-Terpineol	Inactive	0.72	Inactive	0.76	Inactive	0.99	Inactive	0.90	Inactive	0.64	2830 mg/kg	5
9	3-Octanol	Inactive	0.70	Inactive	0.68	Inactive	0.97	Inactive	0.98	Inactive	0.78	1000 mg/kg	4
10	2-Methylphenyl p-methoxybenzoate	Active	0.62	Inactive	0.65	Inactive	0.96	Inactive	0.83	Inactive	0.84	1300 mg/kg	4

Prob (Probability) and T. class (Toxicity class). Class 4: harmful if swallowed (300 < LD_50_ ≤ 2000) and Class 5: may be harmful if swallowed (2000 < LD_50_ ≤ 5000) [34]

## Discussion

Hepatic toxicity is the most well-known organ toxicity, and studies have shown that it is frequently caused by herbal therapy [35]. One of the main signs of hepatic toxicity is abnormal liver function tests. Due to its drug concentration and metabolic activity, the liver is extremely susceptible to damage [35]. The kidney is another organ that is affected by toxic assaults, and it is more vulnerable to blood-borne toxicants than other organs due to its high blood flow, function in urine concentration, and metabolic activation of xenobiotics [35]. The current study investigated whether extracts of *O. lamiifolium* had any toxic effects on the liver and the kidneys of rats after 90 days of the administration, as well as using in silico toxicity prediction methods.

Accordingly, aqueous extracts of *O. lamiifolium* leaves reduced the food consumption and weight gain of the experimental rats. Furthermore, the serum glucose level was significantly reduced in while increment of serum ALT, AST, ALP, and urea was reported. These effects were most noticeable in rats challenged with a high dose (600 mg/kg) of *O. lamiifolium* leaf extracts.

Body weight change is a crucial sign of toxicity, disease development, and therapeutic response [36]. In our study, male and female rats treated with 600 mg/kg bw of the *O. lamiifolium* extract showed decreased food intake and weight gain. The possible justification for this reduced food intake and weight gain could be that *O. lamiifolium* is rich in flavonoids and tannins [21]. Animal studies suggest that flavonoids could reduce fat absorption, boost energy use, and inhibit adipogenesis [37, 38]. Similarly, tannins can harm the gastrointestinal mucosa and reduce food intake [39, 40]. In addition, the essential oil of *O. lamiifolium* contains a significant amount of linalool and this compound is known to inhibit lipid accumulation by down-regulating adipocyte differentiation [41], which could be another potential reason for the reduced weight gain in our rats who received high doses of *O. lamiifolium* leaf extracts.

All experimental rats in the current study did not exhibit treatment-related behavioural changes or other signs of toxicity when clinically observed. Gross examination revealed pyogenic abscesses in the livers of certain rats given high doses of the plant extracts. The increase in liver weight could be due to tumour growth in the liver, which causes hepatomegaly in high-dose aqueous extract *O. lamiifolium*-treated rats [42]. However, treatment with the plant extract did not affect kidneys morphology and weight.

In the present study, the serum level of ALT was significantly increased in male and female rats treated with 600 mg/kg bw of *O. lamiifolium* aqueous extract. Although an increased AST level may not be specific to liver damage, our study found an increased serum AST level in the high-dose treated group (600 mg/kg bw). ALP levels in the blood can rise as a result of bile duct obstruction, liver damage, or bone disease [43]. Our results showed that rats that took 600 mg/kg bw of *O. lamiifolium* extract had higher serum ALP levels. Similar to these findings, a chronic toxicity study of linalool, the primary component of *O. lamiifolium* essential, on mice revealed that AST, ALT, and ALP levels were significantly elevated at high doses [44]. A study suggested that the increment of these enzymes is caused by plant saponins [45] which could explain the observed elevation in liver enzymes in this study too. Another possibility is that *O. lamiifolium* contains compounds that can cause hepatotoxicity. In in-silico toxicity studies, 30% of the total compounds (such as 1-octen-3-ylpropionate, 2-methoxy-4-(1-propenyl)-phenol, and 2-methylphenyl p-methoxybenzoate) were hepatotoxic.

In the present study, treatment with the aqueous extract of *O. lamiifolium* did not affect serum creatinine levels. There was no significant difference in serum creatinine levels between the treatment and control groups of male and female rats. This is consistent with our histological findings which showed none of the treatment groups had anatomical abnormalities in the kidneys. Male rats' serum urea levels did not significantly differ between the treatment and control groups. However, serum urea levels were elevated in female rats given 600 mg/kg bw of *O. lamiifolium* leave extract.

In the current study, serum levels of total protein, albumin, and cholesterol did not differ significantly between rats in the treatment and control groups. Similar findings have been reported in a study on *Ocimum suave* leaf extracts which is a closely related plant species [46].

Blood glucose levels were reduced in male and female rats treated with 600 mg/kg bw of *O. lamiifolium* aqueous extract. This result supported the findings that *O. lamiifolium* had hypoglycemic properties [47]. This could be due to secondary metabolites of *O. lamiifolium* such as terpenoids and flavonoids [21]. Terpenoids and flavonoids have been found to lower blood glucose levels by altering a glucose transporter protein [48].

Rats treated with *O. lamiifolium* leaves extract showed no significant microscopic changes in the normal architecture of the liver and the kidneys in our study. A study on *Ocimum suave*, a closely related plant,also yielded similar results [46]. However, in this study, a small number of female rats treated with 600 mg/kg bw of the extract experienced mild sinusoidal dilatation, central vein dilatation, and parenchymal necrosis. This is most likely due to the effect of compounds identified as hepatotoxic in our *in-silico* toxicity study, such as 1-octen-3-ylpropionate, 2-methoxy-4-(1-propenyl)-phenol, and 2-methylphenyl p-methoxybenzoate.

Along with the in vivo toxicity study performed on animal models, the ProTox-II server [32, 49] was used to assess the toxicity profile of all the constituents of *O. lamiifolium* essential oil. Findings on toxicity and toxicological endpoints demonstrated that none of the compounds derived from *O. lamiifolium* essential oil were cytotoxic or mutagenic. In terms of immunotoxicity parameters, the majority of the compounds (80%) showed no toxicity, while 20% (*δ* -cadinene and trans-*2*-methoxy-*4*-(*1*-propenyl)-phenol) showed immunotoxicity. Furthermore, the majority of the compounds (70%) tested were inactive for carcinogenicity. However, 30% of the tested compounds (linalol, 2,4,5,6,7,7-hexahydro-4,7-methano-1 h-indene, and 2-methoxy-4-(1-propenyl)-phenol) were carcinogenic. Similarly, 70% of the compounds tested were negative for hepatotoxicity. Nevertheless, 30% of the tested compounds were positive for hepatotoxicity. Hepatotoxic compounds include 1-octen-3-ylpropionate, 2-methoxy-4-(1-propenyl)-phenol, and 2-Methylphenyl p-methoxybenzoate. These results are supported by the rise in serum ALT, AST, and ALP levels, which are indicators of liver toxicity and were measured as part of this in vivo toxicity study. Finally, the predicted LD_50_ of the total 10 compounds revealed that 5 compounds (50%) have toxicity class four and are harmful if swallowed, while 5 compounds (50%) have toxicity class five and may be harmful if swallowed [34].

This study provides novel insights into the identification of pharmacologically suitable compounds from leaf extracts of *O. lamiifolium* with future potential applications in clinical medicine.

## Conclusion and recommendations

Our findings demonstrated that oral administration of *O. lamiifoliums* aqueous extract up to the level of 400 mg/kg bw is not harmful. It also had no effect on the histology of the liver and the kidneys. However, in the high-dose (600 mg/kg bw) group, food consumption and weight gain were significantly reduced, while the serum concentration of some enzymes such as ALT, AST, ALP and urea were significantly elevated which may indicate damages to the liver and kidneys. The in-silico results also showed that considerable proportions of compounds were predicted to exhibit hepatotoxic, carcinogenic and immunotoxin properties. Therefore, more scrutiny involving the chronic administration of *O. lamiifolium* extract is ultimately needed to reconcile the discrepancies between functional and histopathological findings. |Besides, before using preparations containing *O. lamiifolium* as a drug, it is necessary to conduct a chronic toxicity study on the essential oil as well as its components that showed toxicity in the *in-silico* toxicity study. Finally, the high doses of *O. lamiifolium* leaves should be used with caution.

## Supplementary Information

**Additional file 1. **

## Data Availability

All the necessary data supporting the result and conclusion of the study have been incorporated into the manuscript.

## Abbreviations

ALP: Alkaline phosphatase
ALT: Alanine aminotransferase
AST: Aspartate aminotransferase
DPX: Dibutyl phthalate Polystyrene Xylene
EPHI: Ethiopian Public Health Institute
OECD: Organization for Economic Cooperation and Development
SDM: Standard deviation of Mean
TMMRD: Traditional and Modern Medicine Research Directorate
